# Beyond a decade: a comparative study of 15-year survival rates in screen-detected vs. symptomatic breast cancer patients in Hungary

**DOI:** 10.1007/s11845-023-03463-x

**Published:** 2023-07-17

**Authors:** Zsolt Varga, Klaudia Balog, Éva Sebő, Péter Árkosy, Dezső Tóth

**Affiliations:** 1 Department of Surgery, University of Debrecen, Debrecen, Hungary; 2 Kenézy Breast Centre, University of Debrecen, Debrecen, Hungary; 3 Department of Oncology, University of Debrecen, Debrecen, Hungary

**Keywords:** Breast cancer, Mammography, Screening, Surgery, Survival

## Abstract

**Background:**

Breast cancer is the leading cancer in women globally. Despite decreasing mortality rates, largely due to early detection and modern treatment, the effectiveness of screening on long-term survival outcomes remains unclear.

**Aims:**

This study evaluates the 15-year survival outcomes of a national breast cancer screening program initiated in Hungary in 2002.

**Methods:**

Using a prospectively maintained patient database, the study included individuals from the first 6 years of the program who underwent surgery for histologically confirmed breast cancer and had available follow-up information. Patients were categorized based on the method of breast cancer detection into two groups: those diagnosed during or 2 years after the population-based screening exam (Group A), and those who self-detected or sought medical attention for symptoms (Group B).

**Results:**

Of the 309 patients who underwent breast cancer surgery, 208 were screen-detected (Group A) and 101 were symptomatic (Group B). The 15-year overall survival was 75.0% for Group A and 76.2% for Group B (*p* = 0.927). The 15-year disease-specific survival was 85.6% and 81.2% (*p* = 0.249), respectively. A statistically not significant positive trend in disease-free survival was observed in Group A (81.7% vs. 75.2%; *p* = 0.144).

**Conclusions:**

The study underscores the importance of extended follow-up periods in evaluating the outcomes of breast cancer screening programs. While the screening program may not significantly enhance overall survival rates, it has demonstrated a reduction in the mastectomy rate and could potentially extend periods of disease-free survival. These findings contribute to the ongoing discourse about the long-term benefits of breast cancer screening programs.

## Introduction

Breast cancer is the most common type of cancer affecting women worldwide with around 2.3 million new cases diagnosed annually, worldwide [1]. While breast cancer remains widespread, mortality rates have been decreasing since the early 2000s. However, despite these improvements breast cancer still resulted in approximately 685,000 deaths in 2020 [2]. The progress in reducing mortality rates is largely due to advancements in early detection and treatment methods. One successful strategy has been the implementation of organized mammography screening programs on a population-wide basis, which have reduced breast cancer-related deaths by approximately 20% by WHO standards [3].

Hungary launched a national breast cancer screening program in 2002, targeting women in the age group of 45 to 65 years old. The plan involved offering biannual screenings to this group of women to detect any early-stage tumors to reduce breast cancer-related morbidity and mortality rates. Our previous study on this program examined short-term outcomes and compared patients identified through screening with those who had symptoms within 6 years from program initiation [4]. A remarkable decrease in mastectomy rates was found within the first 6 years of the program although there has not been any improvement observed in 10-year survival outcomes.

However, data showed that there was a positive trend in disease-free survival among those detected through screening. Consequently, we postulated that extended follow-up periods might reveal even more benefits from this program. Thus, our latest study aims at evaluating 15-year survival outcomes from Hungary’s nationwide breast cancer screening program using our prospectively maintained patient database that includes individuals who underwent surgery for malignant tumors between January 1st, 2002, and December 31st, 2007. Our objective was to broaden our analysis and assess whether there are any substantial oncological benefits associated with organized mammography screening on a population level beyond 15 years after initiation. We aimed to provide a more comprehensive understanding of how population-based breast cancer screening impacts long-term disease prevention and management.

## Materials and methods

Only individuals with confirmed histological breast cancer data and available follow-up information were included in our analysis. When collecting data for this study, we ensured exclusion criteria applied only to those cases featuring recurrent disease, distant metastasis upon diagnosis, or malignancies discovered in different locations. The mammography procedure involved using the GE Senographe 700 T Mammo Unit within the specified period for all patients. For categorization, we considered the method of breast cancer detection: Group A included patients diagnosed during or 2 years after the population-based breast cancer screening exam, which was to ensure the quality of screening by incorporating interval cancers. Meanwhile, Group B sorted individuals aged between 45 and 65 who either self-detected breast cancer or sought medical attention for symptoms.

Clinical data were collected following surgical procedures—including age at surgery, histological and immunohistochemical lesion subtype, primary tumor size as well as pT and pN stage—and thereafter updated based on classification per the eighth edition of the TNM classification of malignant tumors [5]. The short-term study outcomes were previously documented, including the percentages of mastectomy, axillary surgery, as well as adjuvant and neoadjuvant oncologic treatments. The extended oncologic outcomes measured overall survival (OS), disease-specific survival (DSS), and disease-free survival (DFS) 15 years after initial surgery commenced specifically regarding primary tumor surgical treatment. Survival analysis of living patients diagnosed with breast cancer is referred to as OS while DSS is aimed at showing that patients did not experience death caused by breast cancer during the 15-year follow-up period, while DFS refers to the duration from primary treatment until locoregional or systemic recurrence first emerged; this was monitored until May 2023.

All these variables were analyzed concerning both screen-detected age-matched symptomatic patients before significant prognostic factors influencing DSS underwent thorough uni- and multivariate analyses separately. Our statistical analysis entailed IBM SPSS Statistics version 28.0.1.1 (IBM Corp., Armonk, NY, USA). We reported clinicopathologic characteristics in numbers and percentages while using medians with corresponding minimum and maximum values for quantitative variables.

Analysis of variables employed either Pearson’s Chi-squared test or Fisher’s exact test while continuous variable distribution was assessed via the Shapiro–Wilk test and Mann–Whitney *U* tests covered non-normally distributed numerical data. We utilized the log-rank (Mantel-Cox) test to discern differences in survival rates for overall survival (OS) disease-specific survival (DSS) and disease-free survival (DFS) between screen-detected and symptomatic patients. We estimated Kaplan–Meier survivor functions at 180 months for establishing a 15-year survival rate. Cox’s proportional hazards model facilitated multivariable analysis by focusing only on significant associations found via univariate testing. Statistical significance was set as a p-value lower than 0.05. Time intervals were defined as durations from initiation of the first breast cancer therapy to the last event-free visit or an occurrence of locoregional and/or distant relapse or death.

The process of data collection, the revision of histological samples, and the maintenance of the database for this study were conducted under strict ethical guidelines and standards, according to The Declaration of Helsinki. These procedures were approved by the Regional Institutional Research Ethics Committee, Clinical Center, University of Debrecen (approval number: KEK/208/2020.5/2020).

## Results

During the initial 6-year period (2002–2007) following the implementation of the screening program, the average attendance rate was 47.6%, and the recall rate was 4.8%. A total of 309 patients aged between 45 and 65 underwent breast cancer surgery, with 208 patients being screen-detected (Group A) and 101 patients being symptomatic (Group B).

At the time of the surgery, the patients in Group B were significantly older (median age 58.5 years vs 54 years, both range: 45–65 years, *p* < 0.001). No significant differences were observed in terms of the distribution of histologic tumor type, immunohistochemical subtypes of lesions, T-stage, or N-stage. The rate of breast-conserving surgery in Group A was significantly higher compared to Group B (68.8% vs. 59.4%; *p* = 0.032). Up until May 31, 2023, the overall follow-up time had a median of 185 months and a mean of 151.32 months (95% CI = 142.8–159.8). The demographic and clinical data for each group are summarized in Table 1.

**Table 1:** Demographic and clinical features of screen-detected (Group A) and symptomatic (Group B) patients

**Variables**	**Group A**	**Group B**	***p*-value**
Patients (*n*)	208	101	
Age (years)			
Median (min, max)	54.0 (45; 65)	58.5 (45; 65)	0.001
Histology			
IDC	154 (74.04%)	74 (73.27%)	0.879
ILC	19 (9.13%)	12 (11.88%)	
DCIS	15 (7.21%)	6 (5.94%)	
Other	20 (9.62%)	9 (8.91%)	
IHC			
Luminal A	149 (71.63%)	67 (66.34%)	0.285
Luminal B	17 (8.17%)	8 (7.92%)	
HER2-positive	16 (7.69%)	15 (14.85%)	
Triple-negative	26 (12.50%)	11 (10.89%)	
Tumor size (mm)			
≤ 20 mm	134 (64.42%)	57 (56.43)	0.109
>20 mm	74 (35.58%)	44 (43.56%)	
pT stage			
pTis	16 (7.69%)	6 (5.94%)	0.092
pT1	118 (56.73%)	51 (50.50%)	
pT2	69 (33.17%)	36 (35.64%)	
pT3	1 (0.48%)	5 (4.95%)	
pT4	4 (1.92%)	3 (2.97%)	
pN stage			
N0	130 (62.50%)	57 (56.44%)	0.151
N1	58 (27.88%)	25 (24.75%)	
N2	13 (6.25%)	13 (12.87%)	
N3	7 (3.37%)	6 (5.94%)	
Breast surgery			
BCS	143 (68.75%)	60 (59.41%)	0.032
Mastectomy	64 (30.77%)	37 (36.63%)	
Other	1 (0.48%)	4 (3.96%)	
Axillary procedure			
SLNB	18 (8.65%)	6 (5.94%)	0.500
ALND	190 (91.35%)	95 (94.06%)	
Adjuvant chemotherapy			
+	119 (57.21%)	58 (57.43%)	1.000
−	89 (42.79%)	43 (42.57%)	
Adjuvant radiotherapy			
+	170 (81.73%)	79 (78.22%)	0.540
−	38 (18.27%)	22 (21.78%)	
Adjuvant endocrine therapy			
+	153 (73.56%)	73 (72.28%)	0.891
−	55 (26.44%)	28 (27.72%)	
Neoadjuvant therapy			
+	7 (3.37%)	12 (11.88%)	0.005
−	201 (96.63%)	89 (88.12%)	

*IDC* invasive ductal carcinoma, *ILC* invasive lobular carcinoma, *DCIS* ductal carcinoma in situ, *IHC* immunohistochemistry, *BCS* breast-conserving surgery, *SLNB* sentinel lymph node biopsy, *ALND* axillary lymph node dissection

**Fig. 1:**
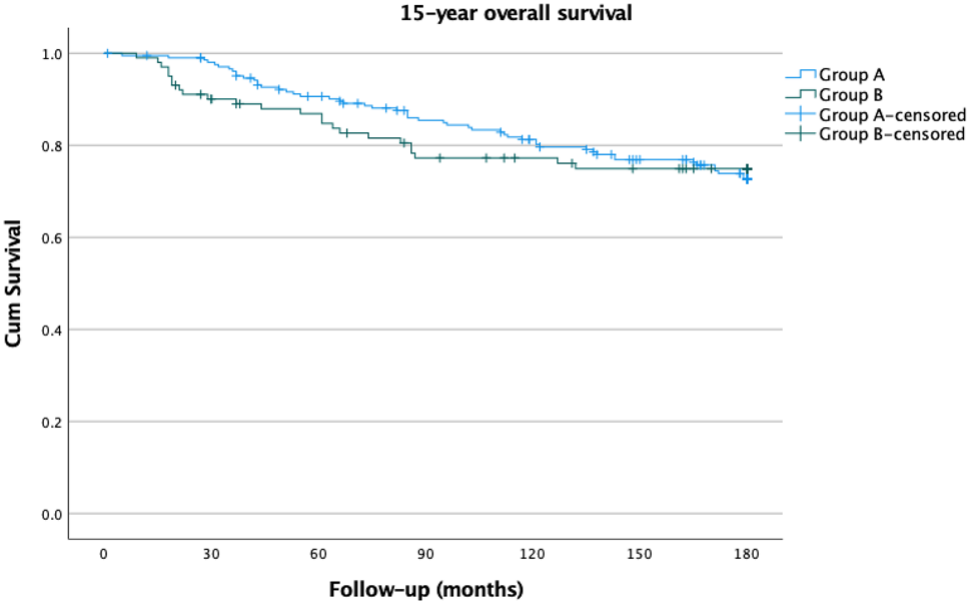
15-year overall survival of screen-detected (Group A) and symptomatic (Group B) patients

**Fig. 2:**
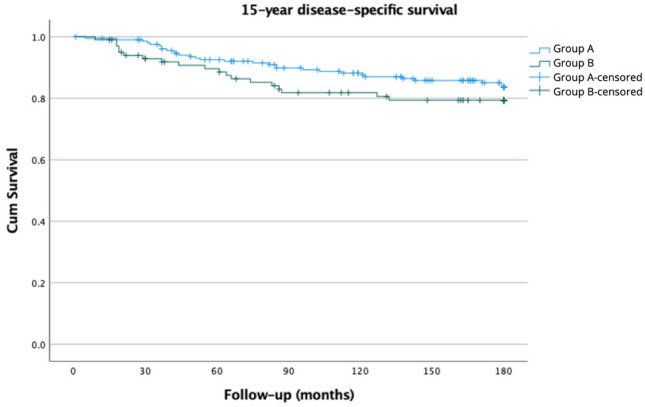
15-year disease-specific survival of screen-detected (Group A) and symptomatic (Group B) patients

**Fig. 3:**
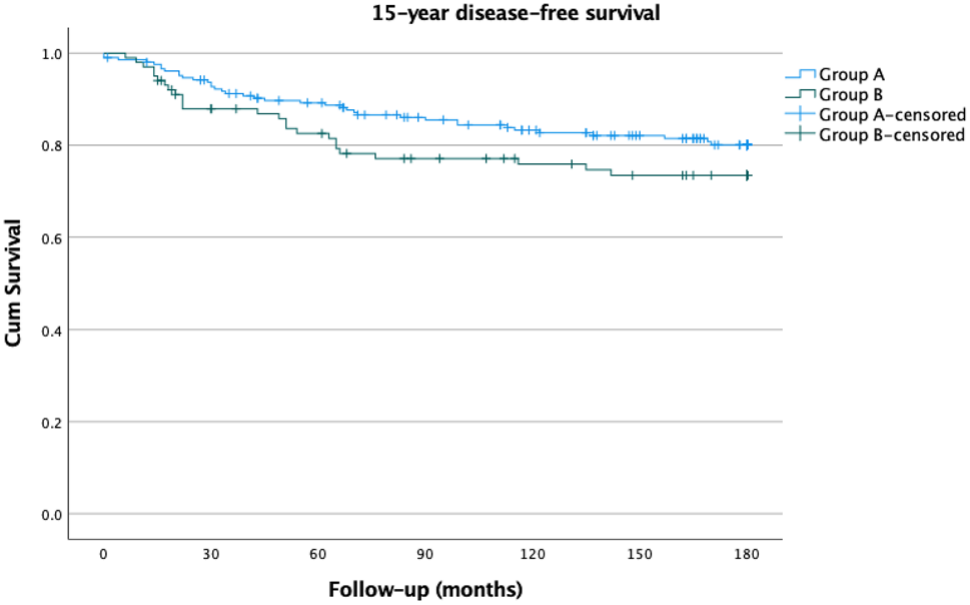
15-year disease-specific survival of screen-detected (Group A) and symptomatic (Group B) patients

The 15-year OS was 75.0% for Group A and 76.2% for Group B (*p* = 0.927) (Fig. 1). Similarly, the 15-year DSS was 85.6% for Group A and 81.2% for Group B (*p* = 0.249) (Fig. 2). As we previously found, there was a positive trend without a significant difference in DFS. The 15-year DFS was 81.7% for Group A and 75.2% for Group B (*p* = 0.144) (Fig. 3). As observed, no survival advantage of screen-detected patients can be demonstrated. Since no subgroup reached a 50% mortality rate, the median survival could not be calculated. We performed univariate analysis to examine the factors affecting the 15-year disease-specific survival. Significant differences were observed concerning histological (*p* = 0.028) and immunohistochemical subtypes of lesions (*p* < 0.001), tumor size (*p* < 0.001), pathological T-stage (*p* < 0.001), and N-stage (*p* < 0.001), type of breast surgery (*p* < 0.001), and the administration of adjuvant (*p* < 0.003) or neoadjuvant chemotherapy (*p* < 0.001) and adjuvant endocrine therapy (*p* = 0.043). The results are summarized in Table 2.

**Table 2:** Univariate analysis of clinical factors on 15-year disease-specific survival

**Variables**	***N***	**15-year DSS (%)**	***p***-**value**
Age (years)			
45–54	154	85.7%	0.321
55–65	155	82.6%	
Histology			
IDC	228	84.6%	0.028
ILC	31	71.0%	
DCIS	21	100%	
Other	29	82.8%	
IHC			
Luminal A	221	91.9%	< 0.001
Luminal B	22	50%	
HER2-positive	27	74.1%	
Triple-negative	39	66.7%	
Tumor size			
≤20 mm	191	92.1%	< 0.001
>20 mm	118	71.2%	
pT stage			
pTis	22	100%	< 0.001
pT1	169	91.1%	
pT2	105	76.2%	
pT3	6	33.3%	
pT4	7	28.6%	
pN stage			
N0	187	91.4%	< 0.001
N1	83	80.7%	
N2	26	61.5%	
N3	13	46.2%	
Breast surgery			
BCS	203	89.7%	< 0.001
Mastectomy	101	75.2%	
Other	5	40%	
Axillary procedure			
SLNB	24	95.8%	0.100
ALND	285	83.2%	
Adjuvant chemotherapy			
+	173	78.6%	0.003
−	136	91.2%	
Adjuvant radiotherapy			
+	243	84.8%	0.418
−	66	81.8%	
Adjuvant endocrine therapy			
+	231	86.1%	0.043
−	78	78.2%	
Neoadjuvant therapy			
+	19	31.6%	< 0.001
−	290	87.6%	

*IDC* invasive ductal carcinoma, *ILC* invasive lobular carcinoma, *DCIS* ductal carcinoma in situ, *IHC* immunohistochemistry, *BCS* breast-conserving surgery, *SLNB* sentinel lymph node biopsy, *ALND* axillary lymph node dissection

When conducting multivariate analysis on these significant characteristics, it was determined that only immunohistochemical subtypes of lesions, pT-, pN-stage, and neoadjuvant therapy remained as independent prognostic factors for 15-year disease-specific survival (DSS). In some cases, the exact value of the hazard ratio was not computed due to the overfitting of the model and the presence of complete separation (Table 3).

**Table 3:** Multivariate analysis of clinical factors on 15-year disease-specific survival

**Factor**	**Contrast**	**Hazard ratio**	**95% CI**	***p***-**value**
Histology				
DCIS	versus IDC	n/a	n/a	0.999
ILC	versus DCIS	2.134	0.962–4.732	0.062
Other	versus DCIS	2.161	0.787–5.935	0.135
IHC				
HER2-positive	versus Luminal A	4.541	1.607–12.836	0.004
Luminal B	versus Luminal A	4.051	1.691–9.703	0.002
Triple-negative	versus Luminal A	5.719	2.145–15.249	< 0.001
pT stage				
pTis	versus pT1	n/a	n/a	0.985
pT2	versus pT1	2.641	1.287–5.418	0.008
pT3	versus pT1	5.520	1.300–23.448	0.021
pT4	versus pT1	6.462	1.413–29.560	0.016
pN stage				
N1	versus pN0	1.550	0.683–3.516	0.294
N2	versus pN0	1.663	0.573–4.832	0.350
N3	versus pN0	10.835	3.567–32.907	< 0.001
Adjuvant chemotherapy				
Given	versus not given	0.897	0.390–2.066	0.799
Adjuvant endocrine therapy				
Given	versus not given	0.926	0.410–2.091	0.853
Neoadjuvant therapy				
Given	versus not given	5.511	2.222–13.664	< 0.001
Screening				
Group B	versus Group A	0.885	0.457–1.715	0.718

*IDC* invasive ductal carcinoma, *ILC* invasive lobular carcinoma, *DCIS* ductal carcinoma in situ, *IHC* immunohistochemistry

## Discussion

Since the 1980s, there has been a significant decrease in breast cancer mortality [6]. A noteworthy factor contributing to this phenomenon in developed countries is the reduced utilization of postmenopausal hormonal treatment following the publication of the Women’s Health Initiative trial, which established a link between such treatment and an elevated risk of breast cancer [7]. Nevertheless, this reduction can mostly be attributed to both the early detection of the disease through screening and advancements in breast cancer treatment methods [8].

Laszlo Tabar and Tibor Tot, two esteemed Hungarian medical professionals, are crucial contributors to the domain of breast cancer screening. Tabar, a distinguished breast radiologist, was the principal investigator in the groundbreaking Swedish Two-County trial, initiated in the 1970s [9, 10]. This trial was the first to prove, with convincing evidence, the life-saving potential of mammographic screening in reducing breast cancer mortality. Tabar’s meticulous work enabled the collection of reliable data and its precise interpretation, effectively revolutionizing our comprehension of early breast cancer detection. The initial publication of the results revealed that mammographic screening correlated with a relative reduction in breast cancer mortality by 31% and stage II + breast cancers by 25%. Consequently, Tabar’s original research significantly shaped international public health policy by setting mammographic screening as the global standard for breast cancer prevention. On the other hand, as a pathologist, Tibor Tot’s work has been crucial in refining pathological techniques and protocols employed in diagnosing breast cancer and predicting prognosis [11, 12]. His contributions have augmented the precision and reliability of these processes, thereby enhancing our knowledge of the disease’s various presentations.

However, a controversial Cochrane review in 2013 pointed out several limitations of the Two-County trial [13]. These included the usage of single-view, single-observer screenings with longer intervals than in other trials, inconsistencies in the cause of death assessment between the trial and the official Swedish death register data, inadequately described randomization processes, and possible non-comparability of randomized groups. Furthermore, regional discrepancies in the proportion of breast cancer diagnoses before the trial’s initiation, variations in the number of randomized women across different publications, and ambiguous timing of the control group’s screening were also mentioned. They posited that the latter may have transpired 5–8 years post-enrollment and noted that cause of death determination was not conducted blindly. This Cochrane review also evaluated existing breast screening trials, determining that the three trials with adequate randomization did not find a statistically significant reduction in breast cancer mortality at 13 years. Nevertheless, the four trials with suboptimal randomization demonstrated a significant reduction in breast cancer mortality, with a relative risk (RR) of 0.75 (95% CI 0.67–0.83). The RR for all seven trials collectively was 0.81 (95% CI 0.74–0.87) [13]. The meta-analysis conducted by the Independent UK Panel on Breast Cancer Screening revealed that the overall relative risk of dying from breast cancer, when comparing invited versus control women, was found to be 0.80 (95% CI 0.73–0.89). It is important to note that most of these trials were conducted several decades ago (1963–1991) when breast cancer treatment was not as effective as it is under current protocols [14]. The benefits of mammography screenings reducing breast cancer mortality tend to become evident after some years emphasizing how long-term follow-up is vital. The WHO position paper suggests that the full impact of mammography screening may become apparent 20 years or more in the future [3].

Our study highlights the necessity and utility of extended follow-up periods when evaluating early detection and treatment strategies for breast cancer patients. The complexities surrounding this disease and its long-term implications make it essential to assess sustained effects over an extended timeframe. In our previous study, we evaluated the short-term and 10-year outcomes of a nationwide screening program in Hungary but found no significant improvement in overall, disease-specific, or disease-free survival rates during this period [4]. First, this conclusion aligns with previous research demonstrating that breast cancer screening programs may not immediately provide their full benefits [15]. Second, while these programs enable earlier detection and treatment, they do not necessarily lead to long-term survival rate improvements. This could be due to many factors, including the nature of the disease, available treatments, and individual patient characteristics. A trend analysis found that although there was a decrease in breast cancer mortality in regions with extensive screening similar decreases were seen in areas without such programs [16]. This suggests that advances in treatment methods are likely contributing to the lack of differences. After 10 years, we did find positive trends toward improved disease-free survival rates for those in the screen-detected group leading us to extend our follow-up period to 15 years for a more comprehensive view of longer-term outcomes. Although this trend is still noticeable, it did not reach the level of statistical significance. In light of these findings, we plan to continue monitoring the outcomes of this cohort of patients for an additional 5 years. This extended follow-up will hopefully provide further insights into the long-term impacts of screen detection on survival outcomes [17].

It was both interesting and encouraging to observe that the vast majority of these patients continued to attend their oncological appointments consistently, even during the global COVID-19 pandemic, which was a serious concern [18]. This underscores the commitment of these patients to their health and the importance of ongoing medical follow-up in managing breast cancer.

In the multivariate analysis, similar to the 10-year outcomes, the immunohistochemical (IHC) subtypes of lesions, pT, pN-stage, and neoadjuvant therapy remain as independent prognostic factors for 15-year disease-specific survival (DSS). This consistency in prognostic factors over time underscores their significant role in affecting long-term survival outcomes for breast cancer patients [19]. It includes the importance of tumor biology in determining the disease’s progression and response to treatment and reaffirms the importance of such well-established indicators as IHC, pT-, and pN-stage in predicting long-term survival.

While our study provides valuable insight into the long-term outcomes of a population-based breast cancer screening program, it is important to acknowledge its limitations. The study’s scope is limited to patients who underwent surgery for a malignant tumor within a specific 6-year period, potentially introducing selection bias and excluding patients treated with non-surgical methods or diagnosed outside this initial period. These findings are based on data from Hungary, which may limit their generalizability to other populations with different healthcare systems, breast cancer prevalence, or screening practices. Since this is an observational study, the lack of randomization to the screen-detected and symptomatic groups could potentially introduce confounding factors. While the 15-year follow-up period is substantial, breast cancer can have late recurrences, and a longer follow-up might reveal additional insights. It must be noted that during this initial period, neither the sentinel lymph node biopsy (SLNB) nor neoadjuvant chemotherapy was standard practice. The introduction of routine SLNB occurred in 2007.

Despite these limitations, we believe our study contributes significantly to the ongoing discourse on the long-term benefits of breast cancer screening programs. Insights from studies like ours will continue to be crucial as we improve our knowledge and refine screening and treatment strategies for confronting breast cancer.

## Data Availability

The data used in this study are available upon request from the corresponding author, subject to any relevant privacy and confidentiality restrictions. Requests for data access should be directed to the authors and will be evaluated on a case-by-case basis, in accordance with the University's policies governing data access and sharing.
